# Nasal polyposis in cystic fibrosis: follow-up of children and adolescents for a 3-year period^☆^

**DOI:** 10.1016/j.bjorl.2016.09.005

**Published:** 2016-10-17

**Authors:** Silke Anna Theresa Weber, Renata Mizusaki Iyomasa, Camila de Castro Corrêa, Wellington Novais Mafra Florentino, Giesela Fleischer Ferrari

**Affiliations:** a Universidade Estadual Paulista “Júlio de Mesquita Filho”, Faculdade de Medicina de Botucatu, Departamento de Oftalmologia, Otorrinolaringologia e Cirurgia de Cabeça e Pescoço, Botucatu, SP, Brazil; b Universidade Estadual Paulista “Júlio de Mesquita Filho”, Faculdade de Medicina de Botucatu, Departamento de Pediatria, Botucatu, SP, Brazil

**Keywords:** Polyposis, Cystic fibrosis, Diagnosis, Endoscopy, Therapy, Polipose, Fibrose cística, Diagnóstico, Endoscopia, Terapia

## Abstract

**Introduction:**

Nasal polyposis is often found in patients with cystic fibrosis.

**Objective:**

To assess the incidence of nasal polyposis, the response to medical treatment, recurrence and the need for surgical intervention in children and adolescents with cystic fibrosis during a three-year follow-up.

**Methods:**

Clinical symptoms (pulmonary, pancreatic insufficiency, malnutrition, nasal obstruction), two positive sweat chloride tests, and genotype findings in 23 patients with cystic fibrosis were analyzed. All patients underwent nasal endoscopy every 12 months from January 2005 to December 2007, to assess the presence and grade of Nasal Polyps. Nasal polyposis, when present, were treated with topical corticosteroids for 6–12 months, with progress being evaluated within the 3 years of follow-up.

**Results:**

In the first evaluation, nasal polyposis was diagnosed in 30.43% of patients (3 bilateral and 4 unilateral), recurrent pneumonia in 82.6%, pancreatic insufficiency in 87%, and malnutrition in 74%. The presence of nasal polyposis was not associated with chloride values in the sweat, genotype, clinical signs of severity of cystic fibrosis, or nasal symptoms. In the three-year period of follow up, 13 patients (56.52%) had at least one event of polyposis, with the youngest being diagnosed at 32 months of age. Only one patient underwent surgery (polypectomy), and there was one diagnosis of nasopharyngeal carcinoma.

**Conclusion:**

The study showed a high incidence of nasal polyposis. Monitoring through routine endoscopy in patients with cystic fibrosis, even in the absence of nasal symptoms, is highly recommended. The therapy with topical corticosteroids achieved good results. Thus, an interaction between pediatricians and otolaryngologists is necessary.

## Resumo

**Introdução:**

A polipose nasal é frequentemente encontrada em pacientes portadores de fibrose cística.

**Objetivo:**

Avaliar a incidência de polipose nasal, a resposta ao tratamento clínico, a recorrência e a necessidade de intervenção cirúrgica em crianças e adolescentes com fibrose cística durante um seguimento de 3 anos.

**Método:**

Os sintomas clínicos (pulmonar, insuficiência pancreática, desnutrição, obstrução nasal), duas pesquisas de cloro no suor positivas e genótipo de 23 pacientes com fibrose cística foram descritos. Todos os pacientes foram submetidos à endoscopia nasal a cada 12 meses durante o período de janeiro de 2005 a dezembro de 2007, para avaliação de presença e grau de polipose nasal. A polipose nasal, quando presente, foi tratada com corticosteroide tópico de 6 a 12 meses, e avaliada a evolução nos 3 anos de seguimento.

**Resultados:**

Na primeira avaliação, a polipose nasal foi diagnosticada em 30,43% dos pacientes (3 bilaterais e 4 unilaterais), pneumonia recorrente em 82,6%, insuficiência pancreática em 87% e a desnutrição em 74%. A presença de polipose nasal não se associou aos valores de cloro no suor, genótipo, sinais clínicos de gravidade da fibrose cística ou sintomas nasais. Nos 3 anos de seguimento, 13 pacientes (56,52%) apresentaram pelo menos um evento de Polipose, sendo o mais jovem diagnosticado aos 32 meses de idade. Apenas um paciente foi submetido à cirurgia (polipectomia), e houve um diagnóstico de carcinoma da nasofaringe.

**Conclusão:**

O estudo mostrou alta incidência de polipose nasal. O acompanhamento por meio de exames endoscópicos de rotina em pacientes fibrocisticos, mesmo na ausência de sintomas nasais, é altamente recomendado. A terapia com corticoide tópico mostrou bons resultados. Sendo assim, faz-se necessária a interação entre pediatras e otorrinolaringologistas.

## Introduction

Cystic fibrosis (CF) is an autosomal recessive disease that affects the exocrine glands, involving multiple organs and progressing chronically and progressively. It is the most common lethal genetic disease in Caucasians, with an average frequency of 1:2000 live births.^1^, ^2^ In Brazil, studies have revealed an incidence of 1:9500 live births in the state of Parana,^3^ 1:8700 in Santa Catarina^4^ and. 1:10,000 in Minas Gerais.^5^

Respiratory infections leading to ultimate respiratory failure are the leading causes of death in CF patients. However, mortality has been reduced in recent years due to earlier diagnosis, greater attention to prophylaxis of recurrent airway infections, and better control of patients in specialized services.^1^, ^2^

CF diagnosis is based on clinical and laboratorial criteria: family history of CF, pancreatic insufficient/pancreatic sufficient, chronic obstructive suppurative lung disease, and two high sweat chloride tests (>60 mEq/L) and/or detection of genetic mutations described in CF. Other clinical data that suggest the disease are: meconium ileus and/or intestinal atresia, hyponatremic dehydration, edema and hypoalbuminemia, chronic panrhinosinusitis, nasal polyposis (NP), volvulus, intussusception, bronchiectasis of unknown etiology, and azoospermia.^6^, ^7^

Upper airway (UAW) impairment such as recurrent rinorhinosinusitis, rhinitis and/or NP occurs in over 90% of patients.^8^, ^9^, ^10^, ^11^, ^12^, ^13^, ^14^, ^15^, ^16^ The incidence of NP, in particular, has been reported in 6–48% patients,^17^, ^18^ and is symptomatic in about 4% patients at diagnosis of CF.^8^, ^10^, ^11^, ^19^ The literature estimates that 14% of patients require surgical treatment of NP.^8^, ^10^, ^11^, ^19^

To date, the pathophysiology of NP is still unknown.^20^, ^21^ Allergic processes have been reported as a possible cause of NP, but the prevalence of atopy in patients with CF is not higher than in the general population.^22^

According to data from the literature and the study previously conducted in our service,^23^ a need for better characterization of the evolution of UAW involvement in these patients was identified.

Thus, the aim of this study was to evaluate, in the medium term, the incidence of NP, the response to medical treatment, the rate of recurrence, and the need for surgical intervention in children and adolescents with CF during a 3-year follow up period.

## Casuistics and methods

The prospective cohort study was approved by the Research Ethics Committee of the institution involved in this research. Parents/caregivers and children over 10 years signed a free and informed consent.

The initial sample consisted of 23 patients (20 males), aged 1 year and 9 months to 22 years and 8 months, followed at the Cystic Fibrosis Reference Center of Pediatrics Pneumology Department of the institution concerned. Epidemiological data (age, gender) and clinical symptoms of CF were obtained, such as meconium ileus, malnutrition, pancreatic insufficiency, recurrent pneumonia and/or other respiratory symptoms, as well as the confirmation of CF through sweat chloride test^7^ and genetic studies. All patients were investigated for complaints of nasal obstruction, mouth breathing, asthma and rhinosinusitis, and underwent nasal endoscopy every 12 months for 3 years. Nasofibroscopic procedures were performed under topical anesthesia with lidocaine spray with no vasoconstrictor. In children under 3 years of age the flexible pediatric nasofibroscope was used (Karl Storz, diameter 2.4 mm), and in the others the rigid nasal endoscope (Karl Storz, 30°, diameter of 2.4 or 4 mm) was used.

The presence or absence of polyps was described, according to the classification suggested by Lund and Kennedy,^24^ in Grade 0 – no polyp, Grade I – polyp in the middle meatus, Grade II – polyp through the middle turbinate, Grade III – polyp filling the entire nasal cavity. During endoscopy, the presence and color of secretion, and nasal mucosa aspect (coloration, edema, degeneration) were evaluated.

Patients diagnosed with NP underwent treatment with nasal topic corticosteroid for six months, and were reevaluated by endoscopy after this period. In case of persistent Polyposis, patients were evaluated with computed tomography of the paranasal sinuses for a possible surgical schedule.

In the statistical analysis, demographic and symptoms data were registered as mean and standard deviation. The association between the presence of polyps and age, sex, clinical symptoms and genetic mutations was assessed by Fisher's exact test, considering significant *p* < 0.05.

## Results

CF diagnosis was confirmed in all subjects through the sweat test. Genetic mutations, using a panel containing 12 mutations, were investigated in all patients, and in 8 patients mutations were detected: ▴ F 508/other, three ▴ F508/▴ F508, one ▴ F508/G 542X, one G542X/other, one R1162X/R1162X, and in 9 patients the mutation could not be determined. A significant proportion of patients had clinical manifestations, including recurrent pneumonia (82.6%), pancreatic insufficiency (87%), malnutrition (74%) and meconium ileus (13%).

The reported respiratory complaints at baseline were asthma in 35% of patients, rhinosinusitis in 22%, and prevalence of oral breathing found in 22%.

**Figure 1:**
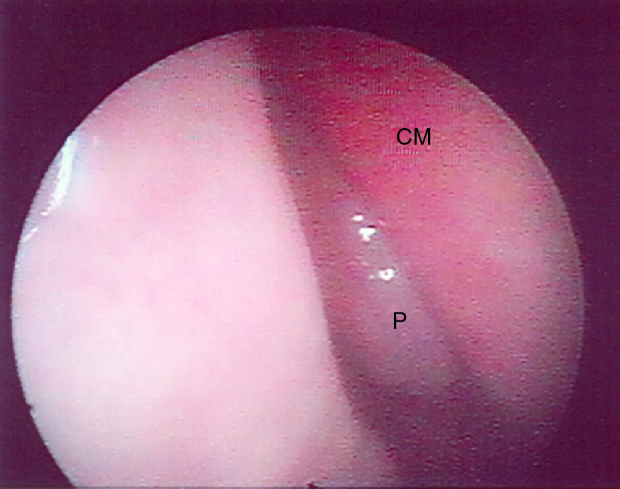
Image of endoscopy of a grade I polyp in the right nasal cavity of patient no. 12 (P, polyp; CM, middle turbinate).

In the first evaluation through nasal endoscopy, Nasal Polyps were found in 7 patients (30.43%). Of these, 3 had bilateral, and 4 unilateral NP, with Grade I in 3 patients, Grade II in 1 patient, and Grade III in 3 patients. No association was found between NP, gender, age, clinical severity, or genetic mutation. Fig. 1 illustrates the results of the endoscopic evaluation (Table 1).

**Table 1:** Results of endoscopy in patients with cystic fibrosis and nasal polyposis at baseline evaluation, first, second and third year of follow-up.

Pat	Gender	Age	Baseline evaluation	Evaluation 1st year of follow-up	Evaluation 2nd year of follow-up	Evaluation 3rd year of follow-up	Behavior
01	F	3 y 8 m	No polyposis	No polyposis	No polyposis	No polyposis	
02	M	8 y 7 m	POLYPOSIS (D – Grade III/E – Grade III)	POLYPOSIS (R – Grade III/L – Grade II)	No polyposis (postsurgery)	No polyposis	Surgery defined on the 2nd year of follow-up
03	M	6 y 4 m	No polyposis	No polyposis	Death	–	
04	M	16 y 4 m	No polyposis	POLYPOSIS (L – Grade I)	No polyposis	POLYPOSIS (L – Grade I)	Topic nasal corticosteroid
05	M	2 y 4 m	No polyposis	No polyposis	POLYPOSIS (R – Grade I/L – Grade II)	POLYPOSIS (R – Grade II)	Topic nasal corticosteroid
06	M	2 y 9 m	No polyposis	POLYPOSIS (R – Grade I)	POLYPOSIS (R – Grade I)	POLYPOSIS (R – Grade I)	Topic nasal corticosteroid
07	M	16 y 2 m	No polyposis	No polyposis	No polyposis	No polyposis	
08	M	3 y 9 m	No polyposis	No polyposis	No polyposis	No polyposis	
09	M	5 y 1 m	No polyposis	No polyposis	POLYPOSIS (R – Grade I)	POLYPOSIS (L – Grade II)	Topic nasal corticosteroid
10	M	3 y 1 m	No polyposis	No polyposis	No polyposis	No polyposis	
11	M	4 y 7 m	No polyposis	POLYPOSIS (R – Grade I)	POLYPOSIS (R – Grade I)	POLYPOSIS (R – Grade I/E – Grade I)	Topic nasal corticosteroid
12	M	8 y 6 m	POLYPOSIS (D – Grade II)	No polyposis	No polyposis	No polyposis	Topic nasal corticosteroid
13	M	3 y 9 m	No polyposis	POLYPOSIS (L – Grade I)	POLYPOSIS (L – Grade I)	No polyposis	Topic nasal corticosteroid
14	M	11 y 7 m	POLYPOSIS (E – Grade I)	No polyposis	No polyposis	Death	Topic nasal corticosteroid
15	M	6 y 3 m	POLYPOSIS (D – Grade II/E – Grade III)	No polyposis	No polyposis	No polyposis	Topic nasal corticosteroid
16	F	8 y 10 m	POLYPOSIS (D – Grade I)	POLYPOSIS (L – Grade I)	No polyposis	POLYPOSIS (L – Grade I)	Topic nasal corticosteroid
17	M	11 y 7 m	No polyposis	No polyposis	No polyposis	No polyposis	
18	M	22 y 8 m	No polyposis	No polyposis	No polyposis	No polyposis	
19	M	3 y 3 m	POLYPOSIS (D - Grade III/E - Grade III)	No polyposis	No polyposis	No polyposis	Topic nasal corticosteroid
20	M	5 y 4 m	POLYPOSIS (E – Grade I)	CA	CA	No polyposis	Topic nasal corticosteroid + chemotherapy and radiotherapy
21	M	14 y 11 m	No polyposis	No polyposis	No polyposis	No polyposis	
22	F	13 y 8 m	No polyposis	No polyposis	No polyposis	No polyposis	
23	M	14 y 0 m	No polyposis	No polyposis	No polyposis	No polyposis	

Pat, patient; Age, referent to baseline evaluation; y, years; m, months; R, right; L, left; CA, nasopharynx carcinoma.

During the 3 years of follow up, 13 patients (56.52%) experienced at least one event of NP, with the youngest being diagnosed at 32 months of age. In these subjects the presence of nasal polyposis was not associated with nasal symptoms, such as nasal obstruction, rhinorrhea or mouth breathing. At the final endoscopy, six patients had polyposis. In all patients, the staging of polyposis was Grade I, indicating lesser severity (*p* < 0.05).

The treatment of NP was nasal topic corticosteroid at the usual dose, and 57.14% of patients responded to medical therapy in the subsequent evaluation, with only one patient who had bilateral NP Grade III not showing satisfactory improvement, and for whom nasal endoscopic surgery was recommended. In these three years, two patients died, and one developed nasopharyngeal carcinoma, undergoing chemotherapy and radiotherapy with a good response. The findings regarding the 3 years of follow-up are shown in Table 2.

**Table 2:** Comparison between younger age with nasal polyposis, number of patients with nasal polyposis, grade of polyposis, unilaterality, presence of surgical indications and complications in the baseline evaluation, in the first, second and third year of follow-up.

	Baseline evaluation	1st year of follow-up	2nd year of follow-up	3rd year of follow-up
No. with NP	7	6	5	6
NP – Grade I (%)	3 (42.86%)	5 (83.33%)	4 (80%)	4 (66.67%)
NP – Grade II (%)	1 (14.28%)	0 (0%)	1 (20%)	2 (33.33%)
NP – Grade III (%)	3 (42.86%)	1 (16.67%)	0 (0%)	0 (0%)
Unilaterality	4	5	4	5
Surgery	0	1	0	0
Complications	0	Nasopharynx carcinoma	1 Death	1 Death

NP, nasal polyposis; No., number of patients; %, percentage.

## Discussion

CF patients’ follow-up in a Reference Center is crucial due to the detection of complications, and the possibility of decision-making by a multidisciplinary team in this service.

The patients evaluated in this study showed classical clinical manifestations of CF, such as meconium ileus, pancreatic insufficiency, malnutrition, and recurrent pneumonia. All patients had diagnostic confirmation supported by two abnormal chloride dosages in sweat, according to a standard method that is supported by literature.^7^

Regarding the result of the detection of genetic mutations, in 52.17% the ▴F508 mutation was present, a high percentage of patients with CF in Brazil, despite the heterogeneity of this population; this corroborates the literature that identifies the association of this mutation with CF.^19^ It should be noted that there was no correlation between the presence or severity of NP and the genotype.

In the literature, NP has been reported with an incidence of 6–48% in CF patients^10^, ^11^, ^25^ In this study, the incidence was 30.43%; it was higher than the one presented in a national study that reported the incidence to be 15.2% in children with a mean age of 9.5 years.^26^ In addition, when monitoring CF patients over a 3-year follow-up, we made the diagnosis of NP in a child of 2 years and 8 months, an age younger than reported in the literature, which describes the occurrence of NP only after 5 years of age.^27^

Even with this study population being predominantly composed of children, there was a high incidence of NP, given that of the 13 cases, 12 were children (less than 12 years) and only one was a teenager of 16 years. The literature brings the incidence of NP of 5% and 15.2% in children.^26^, ^28^

There was incidence of rhinosinusitis and mouth breathing in 22% of patients, similar to that found in the literature.^9^, ^10^, ^18^, ^29^ The presence of NP did not correlate with nasal obstruction or secretion.^18^

Among patients with NP, 3 patients had NP at baseline (42.86%), 5 in 1 year of follow-up (83.33%); 4 in the 2nd (80%), and 4 patients in the 3rd year of follow-up (66.67%) had small polyps, Grade I, highlighting the importance of routine endoscopic examination.^11^ These data exceed the percentage found in literature of 68% of identification of small polyps.^18^

Only one patient required surgery (4.35%), with no recurrence in the subsequent two years; the literature estimates the need for surgery in patients with NP to be 20%^8^, ^9^, ^11^ throughout life. Because of a report of polyp recurrence with a need for surgery in 28.57–58%,^30^, ^31^ these patients require continued monitoring.

Regarding the use of topical corticosteroids, it was observed that 57.14% of patients responded satisfactorily to the initial clinical treatment, and in a subsequent evaluation there was complete involution of the NP, which resembles the information that there is improvement in 56% of patients with NP with topical corticosteroids therapy.^8^ For the population with CF, there is no evaluation data reporting the evolution of NP with clinical treatment for long periods.

We believe that the protocol proposed by this research group of annual endoscopic follow-up of CF patients, in addition to clinical treatment, may be at least partly responsible for the low need for surgical indication. CF is a common, serious genetic disease, but when there is early diagnosis and treatment, comorbidities are reduced, and the quality of life of these individuals improve. The limited number of patients in this study led to difficulties in the statistical analysis, emphasizing the importance of other Cystic Fibrosis Reference Centers also following this protocol and publishing their results in scientific settings.

## Conclusion

The incidence of nasal polyposis in patients with cystic fibrosis is high, even among children, and is not related to the clinical severity of cystic fibrosis or nasal symptoms. Routine annual nasal endoscopy allows early diagnosis of nasal polyps at an early stage (Grade I polyposis), and the initiation of clinical treatment with satisfactory control of the condition. Therefore, the interaction between pulmonologists and otolaryngologists is crucial for the diagnosis, treatment indication, and follow-up of these patients.

## Funding

This study was funded by the São Paulo Research Foundation – 10.13039/501100001807FAPESP (2010/11064–1).

## Conflicts of interest

The authors declare no conflicts of interest.
